# Effect of Service Environmental Parameters on Electrochemical Corrosion Behavior of L80 Casing Steel

**DOI:** 10.3390/ma14195575

**Published:** 2021-09-25

**Authors:** Xiaoguang Sun, Huaiyun Cui, Zhong Li, Renyang He, Zhiyong Liu, Lin Lu

**Affiliations:** 1 Institute for Advanced Materials and Technology, University of Science and Technology Beijing, Beijing 100083, China; sunx_sf@126.com (X.S.); cuihuaiyun@163.com (H.C.); 2 CRRC Qingdao Sifang Co., Ltd., Qingdao 266111, China; 3 Chinese Society for Corrosion and Protection, Beijing 100083, China; zl129616@ohio.edu; 4 Department of Chemical and Biomolecular Engineering, Institute for Corrosion and Multiphase Technology, Ohio University, Athens, OH 45701, USA; 5 China Special Equipment Inspection and Research Institute, Beijing 100029, China; herenyang@163.com

**Keywords:** L80 casing steel, annulus environment parameters, stress corrosion, electrochemical, preloading stress

## Abstract

The corrosion behavior of L80 casing steel was studied in a simulating annulus environment using the electrochemical measurement method, immersion test, and tensile test under a high-temperature and high-pressure H_2_S/CO_2_ environment. The partial pressure of CO_2_ ($P_{{\mathrm{CO}}_{2}}$), the partial pressure of H_2_S ($P_{H_{2}S}$), water content, and preloading stress remarkably affected the corrosion behavior of L80 steel. The influence of $P_{{\mathrm{CO}}_{2}}$ on stress corrosion cracking (SCC) susceptibility has an inflection point of approximately 1.1 MPa. The SCC susceptibility reaches the maximum when the $P_{{\mathrm{CO}}_{2}}$ is about 1.1 MPa. The SCC susceptibility has a positive correlation to $P_{H_{2}S}$ and water content. The higher water content of the corrosion medium can increase the electrical conductivity of the corrosion medium and promote the corrosion of L80 steel, which can improve the diffusion of hydrogen into steel and promote the SCC of L80 steel. Preloading stress can promote local corrosion, thereby promoting SCC of steel under stress. The dislocation emergence point caused by preloading stress can accelerate the diffusion of hydrogen into steel and increase SCC susceptibility.

## 1. Introduction

During oil and gas exploitation, CO_2_ and H_2_S, as associated media, cause serious corrosion damage to oil and gas production equipment, thereby restricting the exploitation of oil and gas fields [1,2,3]. CO_2_ and H_2_S are important influential parameters for oil and gas equipment corrosion. CO_2_ primarily causes electrochemical corrosion, which leads to local pitting corrosion and perforation damage of materials [4,5]. Some researchers have also reported that CO_2_ can cause stress corrosion when dissolved in water [6,7]. On the contrary, H_2_S is more harmful to equipment. H_2_S can cause hydrogen-induced cracking and sulfide stress corrosion cracking, which lead to the breakdown of equipment and can cause huge economic losses and heavy casualties [8,9,10,11].

A huge amount of H_2_S and CO_2_ is found in the annulus environment because of carbonate, sulphate-reducing bacteria, high temperature, high pressure, and other factors [12,13]. In this environment, serious accidents such as leakage and the fracture of casing steel often occur, which leads to the scrapping of the whole oil well [14,15,16]. Casing is the key structure that supports the structural stability and safety of an oil well, and its integrity determines its lifespan. Controlling stress corrosion is important to maintain the safe and stable production of oil wells [4,17]. In the annulus of an oil well, some oil films and scale layers are found on the surface of casing steel because of the presence of mineralized water and a small amount of infiltrated crude oil, which slows down the uniform corrosion to a certain extent [18,19,20]. Therefore, the corrosion of downhole tubes is due to corrosion perforation and stress corrosion fracture under the scale. This case signifies that the sensitivity of stress corrosion of downhole tubes in the annular environment of a production well may be lower than that without crude oil in the annular environment.

In addition, the water content of the corrosion medium changes with the service life of oil well [18,21]. Thus, the SCC behavioral mechanism of the casing changes because of the change of electrical conductivity of the medium. However, a comprehensive understanding of the effect of these factors on the corrosion behavior of tubing in oil wells is lacking.

Designers of oil field generally use two methods for tubing material selection: (1) Select the material according to the mechanical strength of the tubing and completely ignore the stress corrosion cracking (SCC) of the oil well [22]; (2) Select materials according to the working condition of the oil well annulus and NACE TM0177 standard (a test standard for SCC resistance of pressure vessel materials in acidic sulfide environment without considering the inhibition effect of oil film) [23]. However, these methods cannot meet the demand of the oil field [24,25,26]. Therefore, a comprehensive investigation into the corrosion behavior of tubing in the annulus environment of oil fields, as well as the analysis of the mechanism of SCC and the gaining of control of corrosion are necessary, all of which can provide a reference for material selection and prevention.

In this study, the SCC behavior of L80 casing steel was studied by simulating the annulus environment of production wells using the electrochemical measurement method, immersion test, and tensile test under high temperature and high pressure. The effects of partial pressure of CO_2_ ($P_{{\mathrm{CO}}_{2}}$), partial pressure of H_2_S ($P_{H_{2}S}$), water content, and different preloading stresses were studied to fully understand the SCC behavior of L80 steel.

## 2. Experimental

### 2.1. Material and Medium

**Figure 1:**
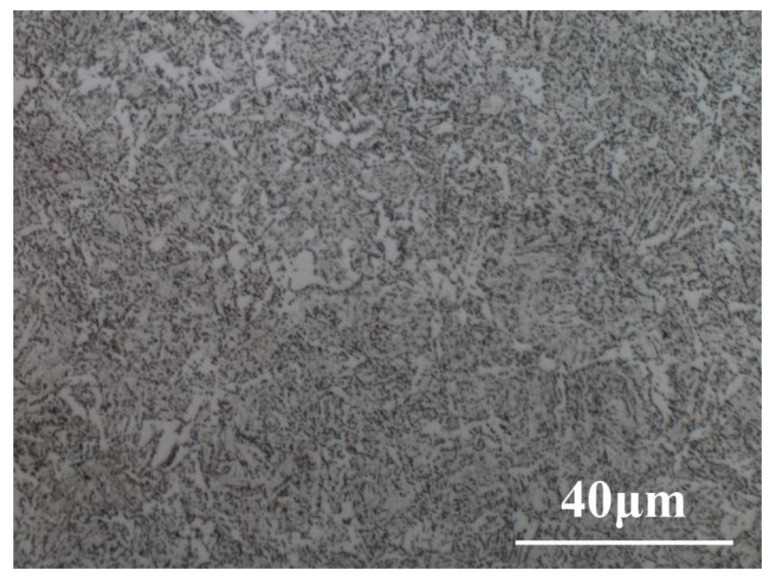
Microstructure of L80 steel.

Specimens were obtained from L80 casing steel. The chemical composition of L80 casing steel is listed in Table 1. The microstructure of casing steels is shown in Figure 1, and the mechanical performance is listed in Table 2. The metallographic sample of L80 steel (Length × Width × Height: 10 mm × 10 mm × 2 mm) was ground using sandpaper of up to #2000 grit. Then, the sample was polished with 0.5 μm diamond paste. Finally, the sample was etched by 4% (volume) nital and observed under metallurgical microscope (VHX-2000, Keyence, Japan). L80 steel was made of tempered sorbite.

According to the analysis of an oil field, the test medium was an oil–water mixture, which was composed of mineralized water and crude oil, the composition of mineralized water was as follows: 236.5 g·L^−1^ of NaCl, 1.01 g·L^−1^ of NaHCO_3_, 0.64 g·L^−1^ of Na_2_SO_4_, 26.64 g·L^−1^ of CaCl_2_, 12.68 g·L^−1^ of MgCl_2_·6H_2_O. The pH of mineralized water was 6.

The water content of the produced medium of oil field was related to the service time of oil well [18]. The proportion of mineralized water (water content) in the test medium was set as 5 wt%, 30 wt%, 50 wt%, 80 wt%, and 100 wt% to simulate the produced medium of an oil field with different service times.

### 2.2. Potentiodynamic Polarization Measurement

Under the service condition of oil casing, the $P_{{\mathrm{CO}}_{2}}$, $P_{H_{2}S}$, and water content of corrosive medium affected the electrochemical corrosion behavior of oil casing. Therefore, potentiodynamic polarization measurement was used to evaluate the effects of the abovementioned factors on corrosion behavior. The potentiodynamic polarization curves were measured under different conditions (as shown in Table 3) by using a three-electrode system in the autoclave. The L80 steel was used as the research electrode, Ag/AgCl electrode as the reference electrode, and platinum plate electrode as the auxiliary electrode. The size of the electrochemical test sample was 10 mm × 10 mm × 4 mm. After welding with copper wire, the L80 steel was sealed with epoxy resin, and the exposed area was 1 cm^2^. The exposed surface of specimens was ground using sandpaper of up to #2000 grit. Then, the specimens were rinsed with ethanol, degreased with acetone, and dried with compressed air. The test temperature was set at 80 °C. The scanning rate of the potentiodynamic polarization test was 0.5 mV s^−1^, and the scanning potential ranged from −0.4 V to 0.7 V (vs. OCP). Before the test, the experimental medium was deoxidized by high-purity nitrogen for 2 h, and the research electrode was polished using 2000-grit sandpapers. After degreasing using acetone, the research electrode was dried for standby. During data processing, all the measured potential were converted into another potential, which refers to the saturated calomel electrode.

### 2.3. Immersion Test

Immersion test was conducted under different conditions (as shown in Table 4). Before the test, specimens were cleaned, dried, and weighed. Then, specimens and test medium were added into the autoclave. Next, the test medium was deoxygenated by purging nitrogen for 2 h. Finally, different experimental conditions were applied, and the immersion test began. All immersion tests lasted for 720 h, and the test temperature was set at 80 °C.

After immersion, all specimens were cleaned, dried, and weighed again. The corrosion rate of specimens was calculated by Equation (1): [27,28]

(1) $$v_{d}=\frac{w_{0}-w_{1}}{\rho \cdot S\cdot t}\times 8,760,000$$

where *v_d_* is the corrosion rate, mm y^−1^; *w*_0_ is the weight of the specimen before immersion, g; *w*_1_ is the weight of the specimen after immersion, g; *ρ* is the density of the steel, which is 7.86 g cm^−3^; *S* is the superficial area of the specimen before immersion, mm^2^; *t* is the time spent by the immersion test, h. All tests were repeated three times for reliable results.

### 2.4. Slow Strain Rate Test (SSRT) after Immersion

**Figure 2:**
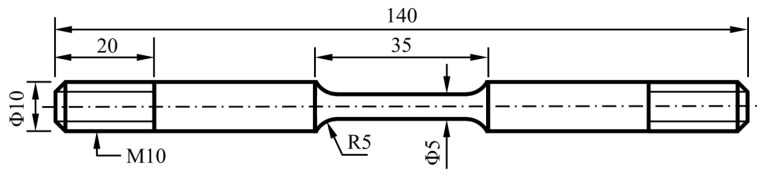
Size of the tensile specimen (unit: mm).

Tensile tests were conducted on other parallel immersed specimens according to ASTM E8M-09 [29]. The size of the tensile specimens is shown in Figure 2. Before the tensile test, the tensile specimens were immersed for 720 h under different conditions (as shown in Table 4). All tensile tests were conducted with a CORTEST slow strain rate test system (CORTEST, Willoughby, OH, USA). The test system can control temperature and gas pressure during tensile test. The tensile rate was 10^−6^ s^−1^. Then, elongations and reductions in the area were obtained from the stress–strain curves. Furthermore, the SCC susceptibility of the L80 steel was calculated by Equations (2)–(4): [30,31,32]

(2) $$I_{\delta }=(1-\frac{\delta_{s}}{\delta_{0}})\times 100\%$$

(3) $$I_{\psi }=(1-\frac{\psi_{s}}{\psi_{0}})\times 100\%$$

(4) $$I_{R_{m}}=(1-\frac{R_{m}{}_{s}}{R_{m}{}_{0}})\times 100\%$$

where *I_δ_*, *I_ψ_*, and *I_R__m_* are the susceptibilities of SCC calculated by different parameters; *δ*, *ψ*, and *R_m_* are the elongations, reduction of area, and tensile strength, respectively; subscript s and subscript 0 represent the parameters of steel in corrosion medium and air, respectively. All tests were repeated three times for reliable results.

## 3. Results

### 3.1. Corrosion Behavior of L80 Steel without Preloading Stress

#### 3.1.1. Effect of Environmental Factors on the Electrochemical Behavior of L80 Steel without Preloading Stress

**Figure 3:**
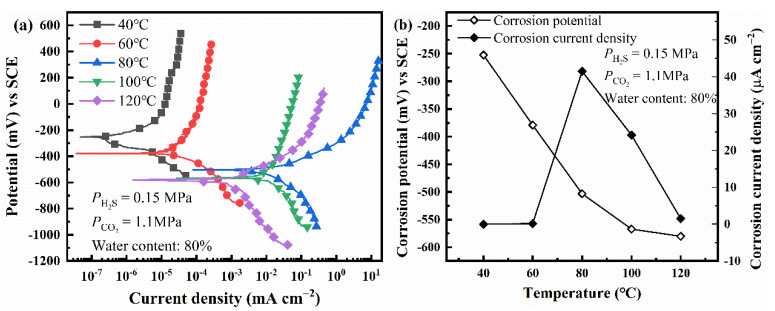
Polarization curves (**a**) and fitting results (**b**) of L80 steels under different temperature.

Firstly, the effect of temperature on electrochemical corrosion behavior is studied using polarization curves, as shown in Figure 3. It can be found that the corrosion potential of L80 steel shifts negatively as the temperature increases. However, the corrosion current density of L80 steel reaches a maximum at 80 °C. Therefore, 80 °C was chosen to research the effect of other factors on the corrosion behavior of L80 steel.

**Figure 4:**
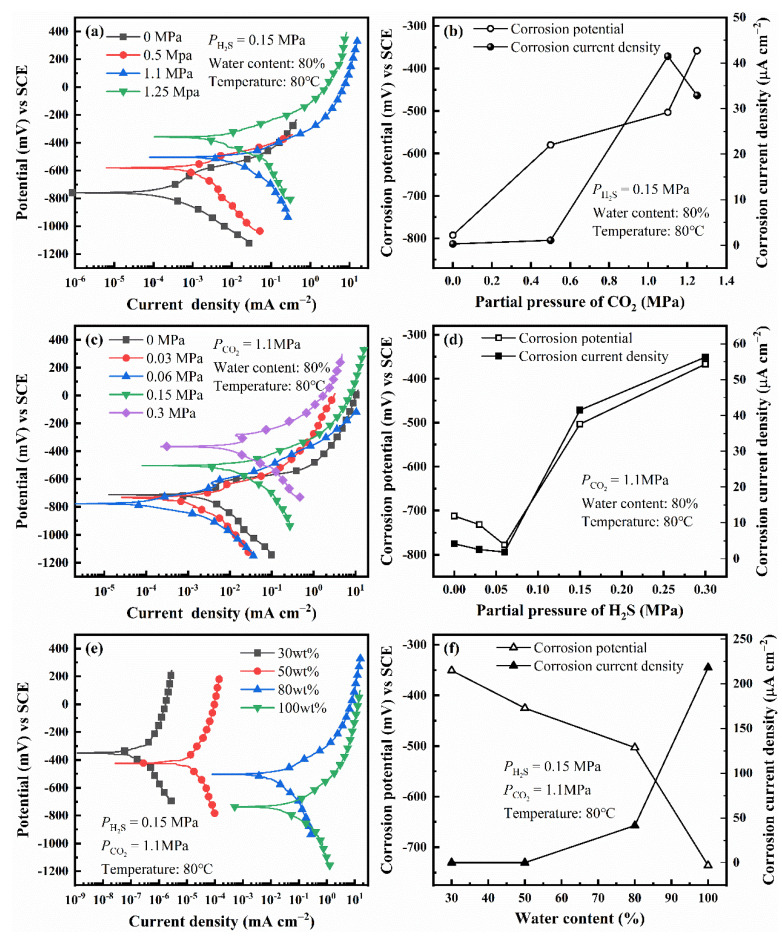
Polarization curves and fitting results of L80 steels under different partial pressure of CO_2_ (**a**,**b**), different partial pressure of H_2_S (**c**,**d**) and different water content (**e**,**f**).

The polarization curves of L80 steel under different experimental conditions are shown in Figure 4a,c,e. Figure 4b,d,f shows the variation of corrosion potential and corrosion current density with different factors, which is obtained by fitting polarization curves. The environmental factors ($P_{{\mathrm{CO}}_{2}}$, $P_{H_{2}S}$, and water content) can affect the polarization behavior of L80 steel. However, when a single factor changes, all the curves have the same shape, which indicates that the variation of a single factor does not affect the corrosion mechanism of L80 steels and the rate-controlling step of corrosion (electrochemical reaction step).

As $P_{{\mathrm{CO}}_{2}}$ increases, the corrosion potential of L80 steel shifts positively; meanwhile, the corrosion current density increases initially and then decreases. The partial pressure related to the largest corrosion current density is called critical pressure, which is 1.1 MPa. The effect of $P_{{\mathrm{CO}}_{2}}$ on corrosion potential and corrosion current density is partially attributed to the change of pH caused by the solution of CO_2_ in the corrosion medium [1,4].

As the partial pressure of H_2_S increases, the corrosion potential of L80 steel shifts negatively first and then shifts positively. In addition, the corrosion current density decreases initially and then increases. Therefore, the increase of $P_{H_{2}S}$ restrains the corrosion of L80 steel in the test medium when the $P_{H_{2}S}$ is less than the critical pressure (0.06 MPa), which may be related to the competitive adsorption of CO_2_ and H_2_S on the surface of L80 steel [28,33].

When the water content changes, the corrosion potential of L80 steel shifts negatively, and the corrosion current density increases with water content. Comparing the four polarization curves under different water content, water content can promote the anodic and cathodic processes of corrosion simultaneously.

**Figure 5:**
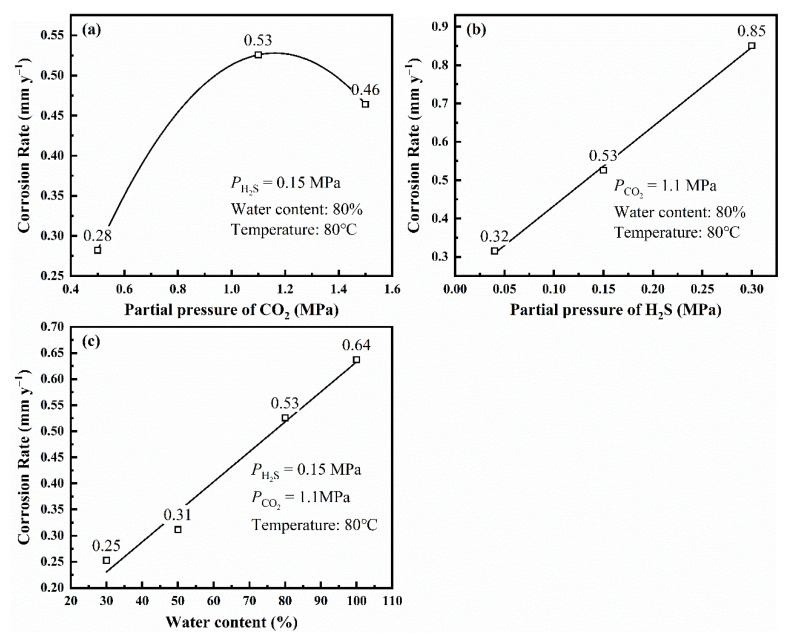
Corrosion rate of L80 steel without preloading stress obtained from immersion test under different partial pressure of CO_2_ (**a**), different partial pressure of H_2_S (**b**), and different water content (**c**).

In verifying the results from the polarization curves, the L80 steel without preloading stress was immersed under different experimental conditions. The corrosion rate of L80 steel was calculated by Equation (1) (Figure 5).

The results from the immersion test are consistent with those from the polarization curves. When $P_{{\mathrm{CO}}_{2}}$ is 1.1 MPa, the L80 steel has the biggest corrosion rate. As $P_{H_{2}S}$ (>0.15 MPa) and water content increase, the corrosion rate of L80 steel under experimental condition increases.

#### 3.1.2. SCC Susceptibility of L80 Steel without Preloading Stress after Immersion

**Figure 6:**
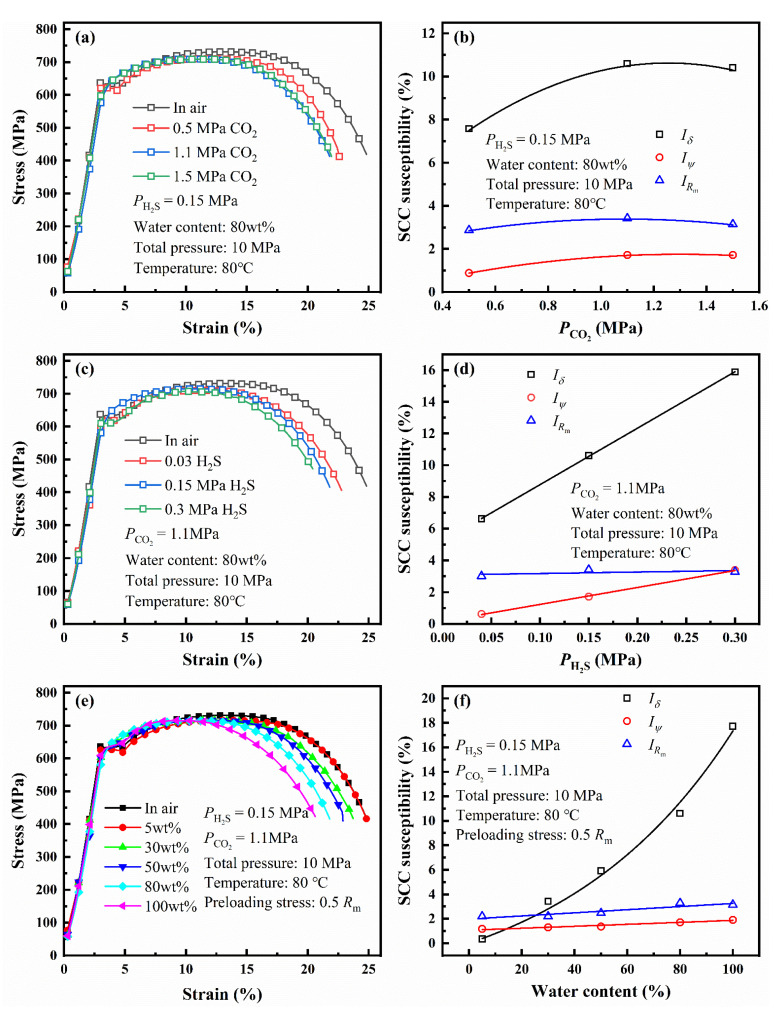
Stress–strain curves and SCC susceptibility of L80 steel under different partial pressure of CO_2_ (**a**,**b**), different partial pressure of H_2_S (**c**,**d**) and water content (**e**,**f**).

Based on the electrochemical behavior of L80 steel, the temperature was set at 80 °C. The $P_{{\mathrm{CO}}_{2}}$, $P_{H_{2}S}$, and water content were changed to study their effect on the SCC susceptibility of L80 steel. Figure 6a,c,e shows the stress–strain curves of L80 steel under different conditions after the 720 h immersion of tensile samples in the test medium.

Figure 6b,d,f shows the variation of the SCC susceptibility of L80 steel with different $P_{{\mathrm{CO}}_{2}}$, $P_{H_{2}S}$, and water content calculated by Equations (2)–(4). Yield strength (*R*_eL_), tensile strength (*R_m_*), and reduction of the area (*ψ*) change slightly as $P_{{\mathrm{CO}}_{2}}$, $P_{H_{2}S}$, and water content increase. *I_Rm_* and *I_ψ_* are all less than 4%, the change of which is too small to reflect the change of the SCC susceptibility of L80 steel [18]. Moreover, *I_δ_* is fitter for analyzing the change of the SCC susceptibility because of the evident change. The value of *I_δ_* is shown in Table 5. The influence of $P_{{\mathrm{CO}}_{2}}$ on *I_δ_* has an inflection point of approximately 1.1 MPa. The *I_δ_* reaches the maximum when the $P_{{\mathrm{CO}}_{2}}$ is about 1.1 MPa. *I_δ_* has a positive correlation to $P_{H_{2}S}$ and water content.

When $P_{{\mathrm{CO}}_{2}}$ is higher than 1.1 MPa, the stress–strain curves and the SCC susceptibility of L80 steel change slightly, which indicates that $P_{{\mathrm{CO}}_{2}}$ slightly affects SCC of steel. However, the variation of the SCC susceptibility of L80 steel with different $P_{H_{2}S}$ is larger than that with different $P_{{\mathrm{CO}}_{2}}$. Therefore, the SCC of L80 in the test medium is more sensitive to H_2_S than CO_2_. Water content also has a great influence on the SCC susceptibility of L80 steel. The SCC susceptibility of L80 steel increases with the water content of the corrosion medium.

### 3.2. Corrosion Behavior of L80 Steel with Preloading Stress

After the downhole tubes are adjusted, the tubes must perform under environmental stress, which can affect the SCC behavior of tubes. Therefore, different levels of initial stress (preloading stress) are applied to the tensile specimen to study how preloading stress affects the corrosion behavior of L80 steel.

**Figure 7:**
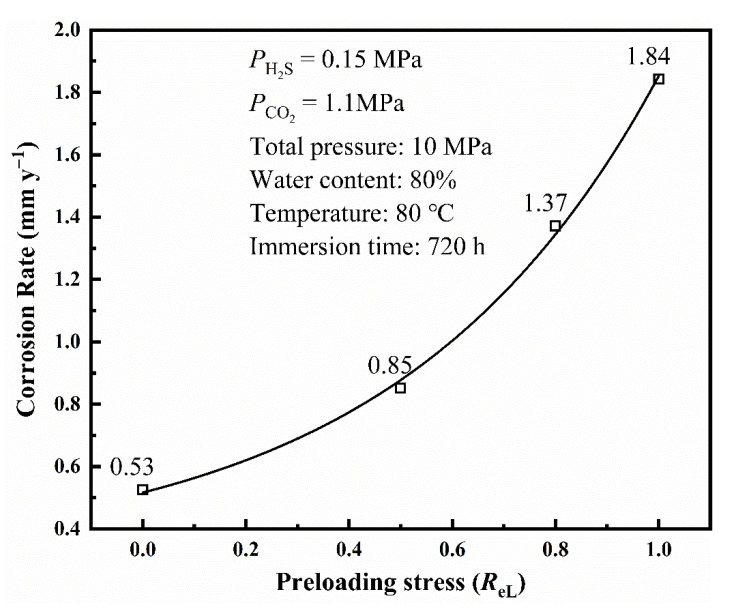
Corrosion rate of L80 steel with different preloading stresses obtained from immersion test.

Figure 7 shows the corrosion rate (calculated by weight–loss method) of L80 steel with different preloading stresses after the 720 h immersion test. The corrosion rate of L80 steel under the experimental conditions increases with preloading stress. Preloading stress can promote the corrosion of L80 steel. The corrosion rate of L80 steel has a positive correlation to preloading stress.

**Figure 8:**
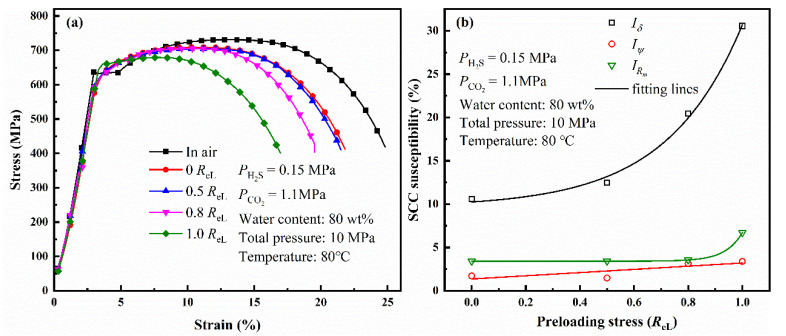
Stress–strain curves of L80 steel with different preloading stresses (**a**) and variation of the SCC susceptibility of L80 steel with different preloading stresses (**b**).

Figure 8a shows the stress–strain curves of L80 steel under different preloading stresses after 720 h immersion. The SCC susceptibility of L80 was calculated using Equations (2)–(4) (Figure 8b). The SCC susceptibility increases with preloading stress. Similarly, *I_δ_* is a good parameter to evaluate SCC susceptibility compared with *I_R__m_* and *I_ψ_*. The value of *I_δ_* is shown in Table 6. *I_δ_* also has a positive correlation to preloading stress, which is similar to the relation between corrosion rate and preloading stress.

## 4. Discussion

When downhole tubes service in a CO_2_/H_2_S environment, the corrosion behavior of tubes is affected by $P_{{\mathrm{CO}}_{2}}$ and $P_{H_{2}S}$, which is complex. After CO_2_ dissociates into the corrosion medium containing water, the following reactions occur: [34,35]

(5) $${\mathrm{CO}}_{2}+H_{2}O⇌H_{2}{\mathrm{CO}}_{3}$$

(6) $$H_{2}{\mathrm{CO}}_{3}⇌H^{+}{+\mathrm{HCO}}_{3}^{-}$$

(7) $${\mathrm{HCO}}_{3}^{-}⇌H^{+}{+\mathrm{CO}}_{3}^{2-}$$

Carbonic acid (H_2_CO_3_) is a weak acid, which can serve as a buffer and keep the pH of the medium at a relatively low value because of the incomplete ionization of H_2_CO_3_ [35]. When H_2_S appears in the solution, reactions similar to Equations (8) and (9) occur, which can also keep the solution acidic [28,36]. The acidic environment can accelerate the corrosion of casing steel. The anodic process is the dissolution of steel (Equation (10)), and the cathodic process is hydrogen evolution reaction (Equation (11)). Hydrogen atoms generated during hydrogen evolution reaction can increase the SCC susceptibility of tubular steels because hydrogen atoms can penetrate the steel substrate [37]. Therefore, every change that can promote hydrogen evolution reaction will increase the SCC susceptibility of tubular steels.

(8) $$H_{2}S⇌{\mathrm{HS}}^{-}+H^{+}$$

(9) $${\mathrm{HS}}^{-}⇌S^{2-}+H^{+}$$

When $P_{{\mathrm{CO}}_{2}}$ increases from 0 MPa to 1.5 MPa, the pH of the solution decreases gradually, and the equilibrium potential of hydrogen evolution reaction shifts positively. The mixed potential of the corrosion system, corrosion potential, is the result of the coupling of metal dissolution reaction and hydrogen evolution reaction. Therefore, the corrosion potential of steel in the corrosion medium increases with $P_{{\mathrm{CO}}_{2}}$ (Figure 4b) [4]. As $P_{{\mathrm{CO}}_{2}}$ changes from 0 MPa to 1.1 MPa, dwindling pH accelerates the corrosion of steel. The further increase of $P_{{\mathrm{CO}}_{2}}$ can increase the concentration of CO_3_^2−^; then, Ca^2+^, Mg^2+^, and Fe^2+^ interact with CO_3_^2−^, which forms insoluble carbonate precipitation (Equations (12)–(14)) [4,6]. Precipitation can cover the anode surface (where the concentration of Fe^2+^ is larger than that on the cathodic area), suppress the anodic reaction, and then decrease the corrosion rate of steel. Corrosion is the coupling of anodic and cathodic reactions [38]. Therefore, the hydrogen evolution reaction is also restricted. The less hydrogen formed on the surface of the steel, the lower the SCC susceptibility of steel [39]. Thus, the results of SSRT (Figure 6a,b) show that the L80 steel achieves the highest SCC susceptibility when $P_{{\mathrm{CO}}_{2}}$ is 1.1 MPa. Figure 3 shows that the corrosion current density of L80 steel reaches a maximum at 80 °C. This is because the higher temperature can improve the corrosion reaction activity and the precipitation of carbonate (FeCO_3_ and CaCO_3_) [40]. When the temperature is lower than 80 °C, the improvement in reaction activity dominates the corrosion process and the corrosion rate increases with temperature. When the temperature is higher than 80 °C, the effect of temperature on the precipitation of FeCO_3_ and CaCO_3_ dominates the corrosion process; the corrosion rate decreases with temperature.

(10) $$\mathrm{Fe}\to {\mathrm{Fe}}^{2+}+2e^{-}$$

(11) $$H^{+}+\;e^{-}\to H$$

(12) $${\mathrm{Mg}}^{2+}+{\;\mathrm{CO}}_{3}^{2-}⇌{\mathrm{MgCO}}_{3}$$

(13) $${\mathrm{Ca}}^{2+}+{\;\mathrm{CO}}_{3}^{2-}⇌{\mathrm{CaCO}}_{3}$$

(14) $${\mathrm{Fe}}^{2+}+{\mathrm{CO}}_{3}^{2-}⇌{\mathrm{FeCO}}_{3}$$

(15) $${\mathrm{Fe}}^{2+}+S^{2}{}^{-}⇌\mathrm{FeS}$$

Under a CO_2_/H_2_S environment, the effect of $P_{H_{2}S}$ on the corrosion of steel differs from that of $P_{{\mathrm{CO}}_{2}}$. Some researchers have reported that the CO_2_/H_2_S ratio can affect the corrosion behavior of steel [4,6,33,41]. The corrosion product scale can be formed on the surface of steel as corrosion develops in a high-pressure CO_2_/H_2_S environment (Equations (12)–(15)) [42,43]. As $P_{H_{2}S}$ increases, the corrosion of the L80 steel is under the mixed control of CO_2_ and H_2_S. The proportion of Fe*_x_*S in the corrosion product film also increases [9].

In the corrosion medium, H_2_S can inhibit the hydrolysis of CO_2_, and the adsorption of H_2_S competes with CO_2_ [28]. Moreover, Fe^2+^ can easily interact with S^2−^ to form precipitation [40]. As the cathode, the precipitation (FeS) can promote the corrosion of L80 steel. When $P_{H_{2}S}$ is less than 0.06 MPa, H_2_S can restrain the corrosion of L80 steel. The corrosion potential shifts negatively, and the corrosion rate decreases. When $P_{H_{2}S}$ is higher than 0.06 MPa, H_2_S also has the abovementioned function (inhibiting the hydrolysis of CO_2_, the adsorption of H_2_S competes with CO_2_, and promoting the precipitation of FeS). However, dissolving enough H_2_S in the corrosion medium promotes hydrolysis and acidifies the medium, which can make up for the inhibited promoting effect of CO_2_ on the cathode process [11]. Therefore, an increase in $P_{H_{2}S}$ can increase the corrosion rate of steel and positively shift the corrosion potential.

Although the corrosion rate of L80 steel decreases and then increases as $P_{H_{2}S}$ increases, the SCC susceptibility of L80 steel always increases as $P_{H_{2}S}$ increases because H_2_S is a poisoning agent, which can increase the hydrogen content in the interior of L80 steel [11]. Hydrogen in the interior of steel can promote stress corrosion.

The water content of the corrosion medium is also an important factor that affects the SCC of L80 steel. As water content increases, the electrical conductivity of the corrosion medium increases. High electrical conductivity can promote the corrosion of L80 steel. In addition, excessive hydrogen can diffuse into the steel and deteriorate the SCC resistance of L80 steel. When the water content is relatively low, the electrical conductivity of the corrosion medium is low, and the corrosion of steel becomes restricted. Moreover, excessive oil can form an oil film on the surface of steel and separate the steel from the corrosion medium, which can also suppress the corrosion of L80 steel.

During the service period, not only the corrosion medium but also the environmental stress affects the corrosion behavior of the L80 steel. In this study, preloading is less than or equal to *R*_eL_. The corrosion rate and SCC susceptibility of the L80 steel both have a positive correlation to preloading stress. When the steel is under an elastic stress state, the stress can lead to dislocation emergence, and a small number of dislocations start to produce defects [44]. These defects increase with the stress level. The dislocation emergence point is a high distortion area with high chemical activity, which can promote corrosion reactions. When stress is equal to *R*_eL_, the dislocations begin to slip. Then, plastic deformation and many defects occur. These defects can also promote the corrosion of steel. Therefore, as the preloading stress changes from 0 *R*_eL_ to 1 *R*_eL_, the number of defects increases rapidly, and the corrosion rate of the L80 steel increases. A higher corrosion rate can produce more adsorbed hydrogen atoms. Furthermore, the lattice distortion caused by tensile stress can improve the diffusion of hydrogen into steel. According to existing research [45,46,47], interstitial atoms (such as carbon atoms) can interact with dislocations and result in dislocation stacking during plastic deformation. However, hydrogen atoms in steel can promote dislocation emission and motion. Next, hydrogen could cause local stress concentration and initiate hydrogen-induced microcracks [48]. Therefore, more hydrogen can diffuse into the steel and promote the SCC of L80 steel.

$P_{{\mathrm{CO}}_{2}}$, $P_{H_{2}S}$, water content, and preloading stress can affect the SCC behavior of L80 steel. A change in these factors can affect the corrosion of L80 steel and the rate of hydrogen evolution reaction. The faster the rate of hydrogen evolution reaction, the more hydrogen diffused into steel, and the higher the SCC susceptibility.

## 5. Conclusions

The results of this study suggest that service environmental parameters ($P_{{\mathrm{CO}}_{2}}$, $P_{H_{2}S}$, water content, and preloading stress) can affect the electrochemical corrosion behavior of L80 steel.

(1) The $P_{{\mathrm{CO}}_{2}}$ affects the corrosion behavior of steel in two aspects: Keeping the pH of the medium at a relatively low value and promoting form insoluble carbonate precipitation. The influence of $P_{{\mathrm{CO}}_{2}}$ on the corrosion rate and SCC susceptibility has an inflection point of approximately 1.1 MPa. The corrosion rate and SCC susceptibility reached the maximum when the $P_{{\mathrm{CO}}_{2}}$ is about 1.1 MPa;
(2) The $P_{H_{2}S}$ also affects the corrosion behavior of steel from two aspects: Inhibiting the hydrolysis of CO_2_ and promoting formation of FeS precipitation. The corrosion rate and SCC susceptibility have a positive correlation to $P_{H_{2}S}$;
(3) Corrosion rate has a linear relation to water content, and SCC susceptibility has a positive correlation to water content. Low water content can decrease the electrical conductivity of the corrosion medium and then restrict the corrosion of L80 steel;
(4) The corrosion rate and SCC susceptibility of L80 steel have a positive correlation to preloading stress. SCC susceptibility of L80 steel can be explained by the local additional potential model. It means that preloading stress can promote the occurrence of defects in the surface of L80 steel. These defects can facilitate local corrosion, accelerate the diffusion of hydrogen into steel, and then increase SCC susceptibility.

## Figures and Tables

**Table 1:** Chemical composition of casing steels (unit: wt%).

Steel	C	Si	Mn	*P*	S	Cr	Cu	Fe
L80	0.29	0.24	1.52	0.010	0.0033	<0.10	0.028	Bal.

**Table 2:** Mechanical performance of L80 steel used in this study.

Steel	Yield Strength *R*_eL_ (MPa)	Tensile Strength *R*_m_ (MPa)	Elongation *δ*_0_ (%)	Reduction of Area *ψ* (%)
L80	633	731	24.8	74.6

**Table 3:** Test conditions of polarization measurement.

Experiment	$P_{{\mathrm{CO}}_{2}}\;(\mathrm{MPa})$	$P_{H_{2}S}\;(\mathrm{MPa})$	Water Content (%)	Temperature (°C)
1-1	0	0.15	80	80
1-2	0.5			
1-3	1.1			
1-4	1.25			
2-1	1.1	0	80	80
2-2		0.03		
2-3		0.06		
2-4		0.15		
2-5		0.3		
3-1	1.1	0.15	30	80
3-2			50	
3-3			80	
3-4			100	

**Table 4:** Test conditions of immersion test.

Experiment	$P_{{\mathrm{CO}}_{2}}$ MPa	$P_{H_{2}S}$ MPa	Water Content %	Temperature °C	Preloading Stress (*R*_eL_)
4-1	0.5	0.15	80	80	0
4-2	1.1				
4-3	1.5				
5-1	1.1	0.03	80	80	0
5-2		0.15			
5-3		0.3			
6-1	1.1	0.15	30	80	0
6-2			50		
6-3			80		
6-4			100		
7-1	1.1	0.15	80	80	0.5
7-2					0.8
7-3					1.0

**Table 5:** The SCC susceptibility of L80 steel under different conditions.

Parameters	Numerical Value				
$P_{{\mathrm{CO}}_{2}}$ (MPa)	0.5	1.1	1.5		
*I_δ_* (%)	7.58	10.60	10.40		
$P_{H_{2}S}$ (MPa)	0.03	0.15	0.30		
*I_δ_* (%)	6.61	10.60	15.88		
Water content (%)	5	30	50	80	100
*I_δ_* (%)	0.36	3.42	5.93	10.60	17.73

**Table 6:** The SCC susceptibility of L80 steel under different preloading stress.

Parameters	Numerical Value			
Preloading stress (*R*_eL_)	0	0.5	0.8	1.0
*I_δ_* (%)	10.60	12.45	20.48	30.55

## Data Availability

Not applicable.
